# Methodological approaches to measuring the incidence of unplanned emergency department presentations by cancer patients receiving systemic anti-cancer therapy: a systematic review

**DOI:** 10.1186/s12874-022-01555-3

**Published:** 2022-03-21

**Authors:** P. H. Dufton, M. F. Gerdtz, R. Jarden, M. Krishnasamy

**Affiliations:** Department of Nursing, School of Health Sciences, University of Melbourne, Carlton, VIC Australia

**Keywords:** Cancer, Systemic anti-cancer treatment, Emergency department, Systematic review, Narrative synthesis, Methodological rigor

## Abstract

**Background:**

The need to mitigate the volume of unplanned emergency department (ED) presentations is a priority for health systems globally. Current evidence on the incidence and risk factors associated with unplanned ED presentations is unclear because of substantial heterogeneity in methods reporting on this issue. The aim of this review was to examine the methodological approaches to measure the incidence of unplanned ED presentations by patients receiving systemic anti-cancer therapy in order to determine the strength of evidence and to inform future research.

**Methods:**

An electronic search of Medline, Embase, CINAHL, and Cochrane was undertaken. Papers published in English language between 2000 and 2019, and papers that included patients receiving systemic anti-cancer therapy as the denominator during the study period were included. Studies were eligible if they were analytical observational studies. Data relating to the methods used to measure the incidence of ED presentations by patients receiving systemic anti-cancer therapy were extracted and assessed for methodological rigor. Findings are reported in accordance with the Synthesis Without Meta-Analysis (SWiM) guideline.

**Results:**

Twenty-one articles met the inclusion criteria: 20 cohort studies, and one cross-sectional study. Overall risk of bias was moderate. There was substantial methodological and clinical heterogeneity in the papers included. Methodological rigor varied based on the description of methods such as the period of observation, loss to follow-up, reason for ED presentation and statistical methods to control for time varying events and potential confounders.

**Conclusions:**

There is considerable diversity in the population and methods used in studies that measure the incidence of unplanned ED presentations by patients receiving systemic anti-cancer therapy. Recommendations to support the development of robust evidence include enrolling participants at diagnosis or initiation of treatment, providing adequate description of regular care to support patients who experience toxicities, reporting reasons for and characteristics of participants who are lost to follow-up throughout the study period, clearly defining the outcome including the observation and follow-up period, and reporting crude numbers of ED presentations and the number of at-risk days to account for variation in the length of treatment protocols.

**Supplementary Information:**

The online version contains supplementary material available at 10.1186/s12874-022-01555-3.

## Introduction

In 2018, there were an estimated 18 million new cancer diagnoses and over nine and a half million cancer related deaths, worldwide [1]. Globally, the incidence of cancer is expected to exceed 27 million new cases per year by 2040 [2]. Cancer treatment often includes localised therapies such as surgery, radiation therapy or systemic anti-cancer therapies (SACT) such as chemotherapy, immunotherapy, targeted therapy and hormonal therapy, and can incorporate a combination of these therapies [3]. Around one in ten patients with early stage cancer, and as many as 45% of patients with later stages of cancer, will receive SACT [4]. The administration of SACT in the inpatient setting is costly and is often reserved for patients who require frequent monitoring. Subsequently, the majority of SACT is administered in the outpatient setting, providing a safe and efficient alternative to inpatient care, which is often preferable to the patient and reduces healthcare costs [5]. Approximately 80% of people who receive SACT will develop treatment related side-effects [6] that can range from mild symptoms that can be easily self-managed at home, to severe symptoms warranting urgent medical care.

Between 10 and 12% of all cancer patients are reported to make an unplanned emergency department (ED) presentation, with approximately 83% of these presentations made by patients undergoing SACT [7, 8]. The receipt of SACT is associated with an increased likelihood of making an unplanned ED presentation [7–10], although there is inconsistent evidence about additional risk factors that are independently associated with ED use [8]. ED presentations by cancer patients are more complex than the general population of ED users; characterised by higher level of acuity, longer length of stay in the ED, significantly higher rate of 28-day re-presentation, and an increased rate of hospital admission, inpatient length of stay, and inpatient mortality [11]. In addition, those who present to the ED are less likely to complete their prescribed SACT than cancer patients receiving SACT who do not present to the ED, potentially compromising treatment outcomes [12].

The need to deliver safe, efficient care to mitigate unplanned ED presentations has received considerable international attention [8, 13–16]. However, in a recent systematic review and meta-analysis by Prince et al. (2019), of a total of 138 studies included in the systemic review and meta-analysis, only 20 contributed to the meta-analysis of the incidence of and risk factors for ED presentation. Authors identified that between 6 to 83% of cancer patients receiving SACT made an unplanned ED presentation [17]. The substantial variance in the reported incidence of unplanned presentations warrants further examination of the methodological approaches used to measure the incidence of ED presentations to inform and strengthen future research and service design to mitigate ED presentations in this population.

## Objectives/research question

The primary aim of this review was to systematically review and examine the methods used to measure the incidence of unplanned ED presentations by patients receiving SACT. Secondary aims include 1) to assess the methodological rigor of studies to determine the strength of the evidence reporting the incidence of ED presentations made by patients receiving SACT, and 2) based on the findings of this review, to offer recommendations for future research investigating the incidence of ED presentations made by patients receiving SACT.

## Methods

The systematic review was conducted according to the Joanna Briggs Institute systematic reviews of prevalence and incidence guidelines [18] and is reported using the Preferred Reporting Items of Systematic Reviews statement [19] and Meta-Analyses Statement Synthesis Without Meta-Analysis (SWiM) guideline [20]. The protocol was registered in PROSPERO (CRD42020162804). Ethics approval was not required for this study.

### Search strategy

Subject headings and keywords from known, available and relevant published articles were used to develop an initial search strategy, which was piloted in the Cumulative Index for Nursing and Allied Health Literature (CINAHL) database. To optimise the search strategy, articles identified as relevant were screened for additional search terms and key words, before the search strategy was translated and was conducted in Medline, Embase and Cochrane databases [21, 22]. Reference lists of included studies were screened for eligible studies. Details of the search strategy are presented in Additional file 1.

#### Eligibility criteria

Search results were limited to studies published in English between January 2010 and December 2019. The date range was limited to the most recent ten years due to the rapidly advancing nature of anti-cancer treatments, techniques and medications used to minimise side effects of SACT, as well as changes to case-mix of patients cared for in outpatient settings. The search strategy was peer reviewed by a librarian with expertise in health sciences and systematic reviews.

#### Inclusion criteria

Included studies were required to report on the incidence of unplanned ED presentations made by cancer patients receiving SACT. Studies were eligible if they met the following inclusion criteria related to study type, participant characteristics, nature of exposure and outcomes:

#### Types of studies

Observational analytical studies that provided data on ED presentations (i.e. the primary outcome of interest for studies included in the review) by cancer patients receiving SACT; cohort, case–control and cross-sectional studies [23]. Observational descriptive studies, reviews, opinion pieces, conference abstracts and articles reporting on research methods only were excluded.

#### Participant characteristics

Studies that reported on adults aged 18 years or above, diagnosed with any cancer and receiving SACT in an outpatient setting. Papers reporting on patients presenting to the ED at the terminal or end-of-life phase, and if studies primarily focused on ED presentations made by cancer patients receiving SACT in the final three months of life were excluded.

#### Types of exposure

Studies that observed participants receiving intravenous and/or oral SACT.

#### Types of outcomes

Studies were eligible if they reported on the incidence of ED presentations.

Screening was undertaken using Covidence [24], an online software program designed to facilitate the process of systematic reviews. Two reviewers (P.D. and C.F) independently screened title and abstracts against the inclusion criteria. Two reviewers (P.D. and C.F) subsequently reviewed the full text of articles that were potentially eligible for inclusion. Any disagreements were resolved by discussion between the reviewers and if necessary, a third reviewer. If additional detail was needed to screen an article, authors were contacted to provide further information.

### Data extraction and quality assessment

Methodological rigor refers to the ‘soundness or precision of a study in terms of planning, data collection, analysis, and reporting’ [25]. Methodological rigor was assessed using the Newcastle Ottawa Scale (NOS). The NOS, developed to assess the risk of bias in non-randomised trials, was used to evaluate the risk of bias of included studies [26]. Cohort and cross-sectional studies were assessed using a version of the NOS adapted specifically for this review. The NOS incorporates three domains, consisting of a total of eight questions. In this review, risk of bias was assessed at the outcome level, that is, ED presentations made by cancer patients receiving SACT. Studies received one star if the study controlled for tumour stream, or type of cancer, and/or stage of disease. Studies were awarded a second star if they controlled for additional demographics such as age, race, and socioeconomic status. These variables were selected based on published literature indicating these factors were likely associated with ED presentations. Both tools are presented in an additional file (see Additional file 2). Studies were considered low risk if they scored three out of a possible three stars; moderate if they scored two; or high if they scored one or zero in selection and exposure/outcome domains. Studies were considered low risk of bias if they scored two out of a possible two; moderate if they scored one; and high if they scored zero in the comparability domain.

All studies were independently evaluated by at least two investigators (P.D. and R.J. or M.K.). The tool was piloted with three studies to compare findings and discuss utility of the tool before the evaluation of all studies was undertaken. Inter-rater agreement scores for risk of bias were calculated as kappa statistics and percentage of agreement using SPSS for Windows (SPSS INC. V26, Chicago, IL.). Data were independently extracted from articles by two investigators (P.D. and K.C.). Any disagreements were resolved through discussion, or if required through a third reviewer. A standardised tool including key study characteristics and methodological rigor was used for data extraction (see Additional file 3).

### Data synthesis

Clinical diversity of studies precluded the ability to synthesise the effect estimates of included studies. To better understand the quality of evidence available, a review of the methodological approaches taken to measure the incidence of studies was undertaken. Data were assessed for methodological rigor and reported using the Synthesis Without Meta-analysis (SWiM) framework [20]. Data were organised for synthesis according to area of methodological rigor of observational studies, that is, study design, population, study setting, and outcome.

As the outcome of interest (i.e. incidence of ED presentations) was reported in multiple formats, where possible, incidence rate was synthesised to calculate the number of ED visits that occurred per unique individual. This method accounts for multiple ED presentations by unique individuals.

Data are presented as tables and findings synthesised in text. The initial synthesis was undertaken by P.D., with further analytical input from M.G., R.J. and M.K.

## Results

### Search findings

**Fig. 1:**
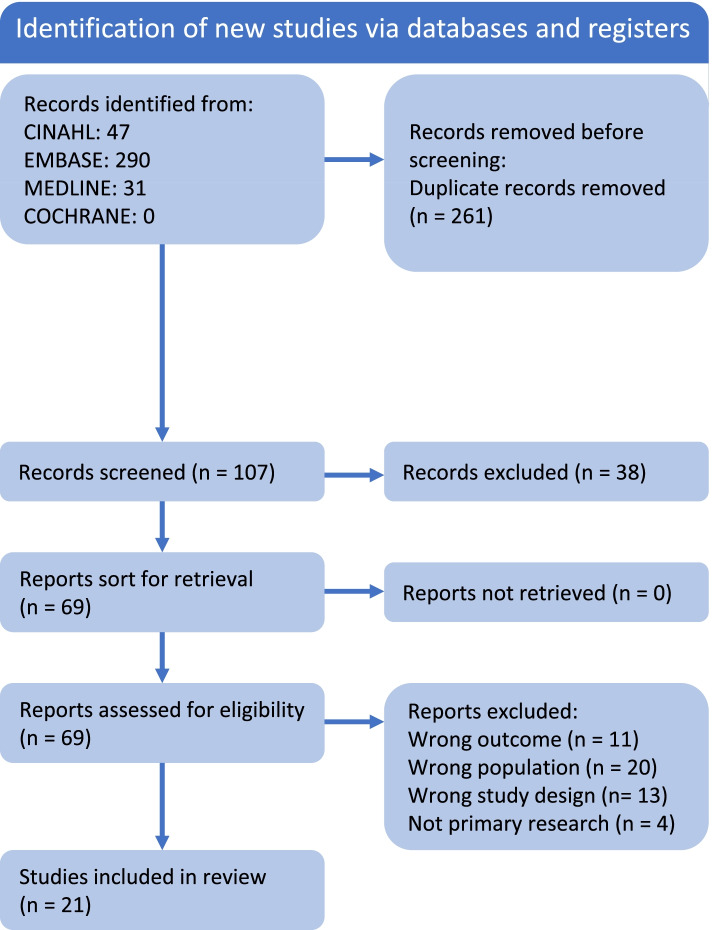
PRISMA Flow diagram

The database search yielded 368 articles, of which 261 were duplicates. One additional study was identified from reference lists, but the article presented the same data as another article potentially eligible for inclusion. After further review, the article identified from the reference list was retained and the initial article excluded [27, 28]. No additional studies were identified by screening reference lists. Sixty-nine articles potentially met the inclusion criteria and were retrieved for full text screening. After review of full text articles, a total of 21 studies were included in the final systematic review (Fig. 1). Twenty of these were cohort studies and one was a cross-sectional study.

### Study characteristics

#### Study design

Of the 21 articles included in the review, most had a retrospective design (*n* = 17, 85%) and were population-based studies (*n* = 11, 52%). Just over a quarter were single site studies (*n* = 7, 33%), and three (14%) were multi-site. Table 1 presents the included study characteristics.

**Table 1:** Characteristics of included studies (*N* = 21)

Authors	Study objective	Study design	Single / multi-site / population	Country	Dates of recruitment	ED presentations per participant case	Risk of bias^b^
Barbera et al. [29]^a^	Evaluate the impact of ESAS screening on ED visits	Retrospective cohort	Population	Canada	Jan 2007—Dec 2009		Low
Eskander et al. [30]	Assess the rate of unplanned hospitalisations and ED visits in the treatment period for patients with HNSCC being treated with curative intent	Retrospective cohort	Population	Canada	Jan 2008—Dec 2012	1.13 ED visits per participant case	Low
Kamat et al. [28]	Report treatment patterns; the source, setting, and division of costs; and survival outcomes	Retrospective cohort	Population	US	Jan 2007—Dec 2011	0.8 ED visits per participant case (1st line treatment) 0.6 ED visits per participant case (2nd line treatment)	Low
Korytowsky et al. [31]	Evaluate HRU rates and total cost of care from a US payer perspective among patients with aNSCLC treated with ST	Retrospective cohort	Population	Not specified	Mar 2013—Dec 2016	0.77 ED visits per participant case (pre-IO) 0.66 ED visits per participant case (post-IO)	Low
Li et al. [32]	Compare medical costs and healthcare resource utilisation for patients using everolimus-based therapy vs chemotherapy	Retrospective cohort	Population	US	Jul 2012—Mar 2014	0.10 ED visits per person (line 1)	Low
Mehra et al. [33]^a^	Analyse patterns of healthcare resource utilisation and costs among a large group of commercially-insured patients receiving docetaxel for APC	Retrospective cohort	Population	US	Jan 2003—Dec 2009		Low
Williams et al. [34]^a^	Evaluate concordance of treatment regimens received (hormone therapy, chemotherapy, and targeted therapies) with NCCN guidelines for older women with early-stage breast cancer; understand categories of discordant treatments; and determine the impact of discordance on health care utilisation (hospitalisations and ED visits) and spending	Retrospective cohort	Population	US	2012—2015		Low
Colligan et al. [35]^a^	Estimate the impacts of two programs and assess their relative effectiveness to inform future models to reduce utilisation and spending in cancer patients	Retrospective cohort	Multi	US	2010—2015		Moderate
Dufton et al. [36]	Explore socio-demographic [46] and disease related characteristics of cancer patients presenting to an ED within 28 days of receiving infusional, systemic anti-cancer therapy in a Day Oncology Unit, in order to identify those at greater risk of making an unplanned ED presentation	Retrospective cohort	Single	AUS	Dec 2014—Nov 2017	0.88 ED visits per participant case	Moderate
Enright et al. [37]	Examine the frequency of ER&Hs associated with chemotherapy	Retrospective cohort	Population	Canada	Jan 2007—Dec 2009	0.35 ED visits per participant case	Moderate
Fisher et al. [38]	Compare health care resource use, treatment patterns, and economic outcomes among patients with cancer receiving intravenous chemotherapy	Retrospective cohort	Population	US	Jan 2006—Aug 2012	0.3 ED visits per participant case (treated in physician office) 0.8 ED visits per participant case (treated in hospital outpatient setting)	Moderate
Harrison et al. [39]	Assess the frequency and severity of patient-reported, chemotherapy- related toxicities in adults receiving their first cycle of chemotherapy for cancer, compared with the frequency of toxicities documented by clinicians. Second, assess factors are associated with unplanned health care service use resulting from toxicities reported by patients after first-cycle chemotherapy	Cross-sectional	Single	US	Not stated	NA	Moderate
Minami et al. [40]^a^	Clarify the frequency with which Japanese lung cancer patients visit the ED after hours	Retrospective cohort	Single	Japan	Jan 2008—Jun 2012		Moderate
Peyrony et al. [41]	Estimate the frequency with which patients with cancer undergoing ICB at our institution present to the ED	Retrospective cohort	Single	France	Jan 2012—Jun 2017	NA	Moderate
Hoverman et al. [42]	Evaluate the impact of Innovent on Level I Pathway compliance within TXO, implement PSS, measure the rate and costs associated with chemotherapy-related ER visits and in-patient use, and assess chemotherapy costs	Prospective cohort	Population	US	Jun 2010—May 2012	0.21 ED visits per participant case (baseline) 0.11 ED visits per participant case (Innovent)	High
Pittman et al.[43]	Analyse the reasons why patients present to the ED and to determine what factors are associated with ED visits and hospital admissions after curative chemotherapy for breast cancer	Retrospective cohort	Single	Canada	2011 and 2012	0.59 ED visits per participant case	Moderate
Schwartzberg et al. [44]	Compare the incidence of CINV and its associated healthcare resource utilisation with NK1 receptor antagonist or non-NK1 receptor antagonist	Retrospective cohort	Population	US	Jun 2013—Dec 2013	0.19 ED visits per participant case	Moderate
Tang et al. [12]	Study ED usage by early breast cancer patients undergoing chemotherapy in an Australian academic hospital setting, and to describe factors associated with ED presentations and hospital admissions	Retrospective cohort	Single	AUS	Jan 2014—31 Dec	0.62 ED visits per participant case	Moderate
Ward et al. [45]	Describe the proportion of monthly treatment costs borne by four major financing agents and identify the monthly costs for each element of health care in this cohort	Prospective cohort	Multi	AUS	Jan 2009—Oct 2010	0.74 ED visits per participant case	Moderate
Baenda-Canada et al. [46]	Analyse need for extraordinary health-care attention	Prospective cohort	Single	Not specified	Jan 2007—Mar 2011	0.14 ED visits per participant case	High
Livingstone et al. [47]	Assess the characteristics of unplanned ED presentations and care pathway	Retrospective cohort	Multi	AUS	Jan 2007—Dec 2007	0.57 ED visits per participant case	High

^a^ED presentations per participant case unable to be calculated as study only reports the number of unique individuals that made on or more ED presentation and not the total number of ED presentations made

^b^Newcastle Ottawa Scores correspond to high, moderate or low according to the number of stars received as 0–4, 5–6, or 7–8, respectively

*Abbreviations*: *ESAS* Edmonton Symptom Assessment Scale, *ED* Emergency Department, *ER&H* Emergency Room and Hospitalisation, *HNSCC* Head and neck squamous cell carcinoma, *TXO* Texas Oncology, *PSS* Patient Support Services, *HRU* Healthcare resource utilisation, *aNSCLC* Advanced non-small cell lung cancer, *ST* Systemic antineoplastic therapy, *APC* Advanced prostate cancer, *ICB* Immune checkpoint blockade, *CINV* Chemotherapy induced nausea and vomiting, *NK1* Neurokinin-1, *NCCN* National Comprehensive Cancer Network, NA - Not applicable

### Quality appraisal

**Fig. 2:**
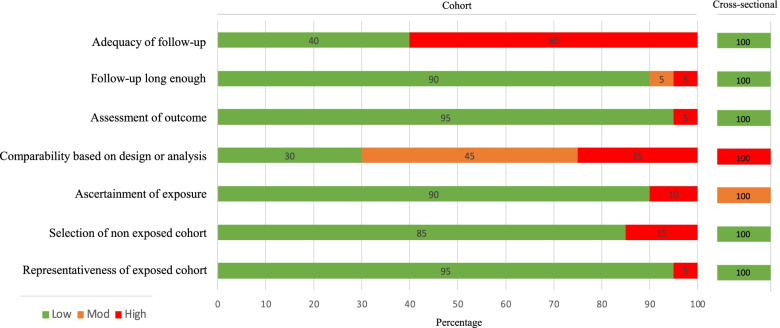
Critical appraisal questions in 21 studies using the Newcastle–Ottawa Scale

There was good agreement for quality appraisal between the two assessors (k = 0.759, *p* < 0.001, percentage agreement = 88%) [48]. Results of the quality appraisal for the 20 cohort studies and single cross-sectional study are presented in Fig. 2. The number awarded to the 20 cohort studies ranged from two to eight of a possible eight. The cross-sectional study received six out of a possible eight [39]. Nine studies (5%) were limited by not providing information about participants who were lost to follow up during the study period [12, 29, 34, 36, 37, 41–43, 46, 47]. In a total of 15 studies (75%), it was not possible to compare participants who presented to the ED with those that did not due to a lack of information or control during statistical analyses (six scored zero [38, 39, 41, 42, 45, 47], and nine scored one [12, 28, 33, 35, 36, 40, 43, 44, 46]).

#### Setting

Most of the studies were undertaken in the US (*n* = 9, 43%), Australia (*n* = 4, 29%), and Canada (*n* = 4, 20%). Dates of recruitment ranged from 2003–2017. Six studies (39%) described the health service and usual care provided to support outpatients receiving SACT, who experienced side-effects of treatment [35, 36, 40, 42, 46, 47]. Four studies (19%) reported availability of an enhanced model of care whereby participants had access to a dedicated cancer nurse as well as phone support during and after hours [36, 42, 46, 47].

#### Methods

Fourteen studies (67%) enrolled patients at diagnosis or initiation of SACT [29–34, 37, 39, 40, 42, 44, 46, 47], with six studies (29%) including all individuals with ongoing treatment during the set study dates [12, 35, 36, 41, 43, 45]. The period of follow-up was defined in 11 (52%) studies [28, 30, 33–35, 39, 40, 44, 45], and a summary of the time participants were followed up was reported in eight studies (38%) [28, 30, 31, 33, 38, 42, 44, 45]. With the exception of the study by Minami et al. that followed patients from death through to diagnosis, only two studies (10%) [35, 38] reported the proportion of patients that died during the study period. Importantly, no studies provided details about participants lost to follow-up. See Additional file 4 for data.

Seven studies (33%) reported a period of observation from the receipt of SACT to ED presentation of 28–30 days [12, 33, 35–37, 42, 43]. Other studies reported a period of between six and 15 months (*n* = 4, 19%) [30, 38, 45, 47], with one study (5%) reporting a five-year period of observation [41]. Six studies (29%) reported the period of observation as ‘during treatment’ [28, 29, 34, 39, 40, 44]. Three studies (14%) made no statement about the period of observation [31, 32, 46].

#### Participant characteristics

The 21 studies included a total of 72,904 participants. The combined mean age across all studies ranged from 46–73 years. Cancer type and stage of disease varied across studies.

#### Exposure

Most studies (*n* = 20, 95%) reported participant exposure to various chemotherapy agents, with only one study limiting inclusion to patients receiving docetaxel chemotherapy only [33].

#### Outcome measures

Six studies (30%) reported that ED presentations recorded were ‘all-cause’ and included all ED presentations made by cancer patients that fell within the period of observation [32, 33, 38, 40, 41, 44]. Five studies (25%) recorded ED presentations that were defined as specifically relating to treatment; one study (5%) [37] used a previously developed algorithm used to identify chemotherapy related adverse events in breast cancer patients [10], and two studies reported on a priori defined common adverse events related to SACT [28, 37]. Cause of ED presentation was determined by a single investigator in one study [46], and in another, cause was self-reported by participants [39]. Whether ED presentations were all cause or SACT related was not stated in ten studies (48%) [12, 29–31, 34–36, 43, 45, 47]. Emergency department presentations were classified differently between studies. For example, Eskander et al. [30] and Schwartzberg et al. [44] classified ED presentations as a single, mutually exclusive event, meaning that if the patient was hospitalised following an ED presentation, this event was classified as a hospitalisation only. Conversely, Peyrony et al. [41] only collected data on the first ED presentation that occurred for every patient during the observation period.

#### Potential confounders

Most studies controlled for cancer type (*n* = 17, 81%) [9, 12, 28–34, 36–40, 42, 46] and stage of disease (*n* = 17, 81%) [9, 12, 28–35, 37, 39, 40] as potential confounders in study design. Other potential confounders controlled for during design or analysis included age (*n* = 10) [29–31, 34–39, 46], gender (*n* = 13) [9, 12, 29–32, 34–39, 46], race (*n* = 3) [9, 34, 35], rurality (*n* = 7) [9, 29–31, 37, 38, 40], comorbidities (*n* = 8) [9, 29, 31, 32, 34, 37–39], socioeconomic status (*n* = 4) [29, 30, 36, 37].

#### Statistical analyses

Most studies use a logistic regression model to adjust for potential confounders. Only two studies [29, 36] reported the methods used to adjust for repeated measures, as ED presentations are likely to occur multiple times for any individual, and subsequently events that occur in the same subject will be intrinsically correlated [49]. For example, Barbera et al., [29] utilised a recurrent event model to account for likelihood of the outcome to occur multiple times during the observation period. Conversely, Korytowsky et al. [31] reported predicators of total costs related to health service utilisation using multivariate logistic regression. Details of methods to adjust for recurring events, and hence, avoid correlation were not reported. Most studies included variables likely to be affected by time, as time-fixed variables. For example, in studies that observed participants over substantial periods of time, covariates such as age, stage of disease and comorbidities will change.

#### Study results

The number of ED presentations per participant case was able to be calculated in 14 out of 21 studies, with the number of ED presentations ranging from 0.10 to 1.13 per case. However, this range represents varying time periods of observation, in different cohorts, and ED presentations for different reasons. Previous literature suggests that the incidence of ED presentations in a similar cohort does not occur at a constant rate, but occurs at greater incidence during the initial SACT treatments [50]. The number of ED presentations per participant cases are presented in Table 1.

## Discussion

This systematic review sought to review the methods used and to assess the methodological rigor of observational studies reporting the incidence and prevalence of ED presentations by patients receiving SACT. Details of methodological approaches identified in this review are summarised in additional file 4. The diversity of methods used to measure incidence and prevalence of ED presentations, as well as the methodological rigor of studies and potential biases introduced are discussed below.

### Internal validity

#### Study design

Most studies (*n* = 17) included in this review used a retrospective study design. In the paper by Harrison et al. [39], a prospective cohort study design was used to explore unplanned health service use by patients after receiving the first cycle of chemotherapy. A strength of this study, enabled by the prospective design, was the range and precision of data collected. This included detailed treatment and demographic data, which allowed researchers to measure and control for potential confounders associated with the outcome of interest [51]. However, limitations include the small sample size, and taking nine months to recruit 100 participants across five centres. Prospective study designs may also be hampered by observer bias, or Hawthorne effect, where participants in a study alter their behaviour as a consequence of being aware that their behaviours are being studied [52]. Prospective studies also have potential for selection bias, whereby certain participants will volunteer to participate, and others will not. Findings from a systematic review by Kho et al. [53], demonstrated that factors such as age, race, income, education and health status may vary among participants who do and do not consent to take part in research studies.

Seventeen papers in this review used retrospective approaches which present different methodological limitations. For example in the retrospective cohort study by Dufton et al. [36], it was not possible to collect a potentially important confounder, stage of disease, as it was not routinely collected in the administrative healthcare datasets at the study site. While retrospective cohort studies allow for inclusion of a greater breadth of events or variables of interest than prospective study designs, and subsequently are more representative of the larger population or group of interest, they lack control over definitional precision or standardisation of variables collected, presenting a major limitation to the quality of findings generated by retrospective study designs.

#### Outcome definition/measurement

Fourteen studies reported that enrolment (that is, time point of entry to the dataset) was triggered by diagnosis, first line treatment, or death. Enrolment at diagnosis, or first line treatment helps to account for prior exposure to other anti-cancer treatments. If enrolment is defined by study dates rather than a specified time period of follow up, there is potential to miss outcomes relating to an unplanned ED presentation that occurs in participants who enter the dataset towards the end of the data collection time period.

There was also substantial variation in how the studies measured unplanned ED presentation as the outcome of interest. For example, Baenda-Canada [46] included ED presentations that were only related to symptoms associated with SACT. Furthermore, whether or not the ED presentation was related to treatment was determined by a single study author without any independent review. This may have led to misclassification bias whereby ED presentations are either incorrectly included or excluded. Two of the included studies used an existing algorithm developed by Hassett and colleagues, [10] to standardise identification of treatment-related ED presentations only among breast cancer patients.

The period of observation varied greatly across the studies reviewed, resulting in potential to over- or under-estimate the incidence of unplanned ED presentations associated with SACT [17]. Another important consideration is the period of follow up across each of the included studies. Follow up time periods ranged from one month [44] to three years [35]. It is important to recognise that the longer the time period of follow up, the more opportunity there is for the outcome of interest to occur (irrespective of whether associated with use of SACT or not) and as such, for conclusions about the incidence of unplanned ED presentations among outpatients receiving SACT to be inaccurate.

#### Loss to follow-up

In prospective studies, attrition bias occurs when an investigator loses contact with participants, resulting in missing data. In retrospective study designs, attrition bias refers to participants who exit the dataset because of discontinuation of treatment or death, for example [54, 55]. Loss to follow up is rarely random and differences between those that drop out or are lost to follow up, and those who remain in the study, must be examined to determine if there are any significant differences that may impact study findings [56]. One of the risks when observing people affected by cancer is the substantial risk of loss to follow-up because of deteriorating health or disease progression. For example, in the retrospective cohort study by Li et al. [32], health resource use in women with metastatic breast cancer was explored over a two-year period. In the cohort of participants treated with SACT, almost 50% of participants had less than three months of follow-up data. This suggests that participants either discontinued treatment or died within three months of entering the dataset. Generally, if attrition is greater than 20%, this has potential to impact the internal validity of a study [57]. Importantly, no studies included in this review provided a detailed description of patients who were lost to follow-up during the study period. As loss to follow up is likely related to the outcome or the exposure, that is, patients may be hospitalised for extended periods of time or die due to their cancer after making an unplanned ED visit, this has the potential to affect internal validity.

#### Confounding

Confounding is where the risk of the outcome occurring within exposed and unexposed study groups is influenced by an alternative or unaccounted for variable [51]. Potential confounders include factors that are known to be associated with the outcome based on prior knowledge of the literature. The majority of studies included in this review controlled for tumour type, either by limiting the population included in the study design or through statistical analysis. Just under half of the studies (*n* = 9, 43%) included in this study included stage of disease as a potential confounder. Patients with advanced or metastatic disease may present with complex disease related symptoms and subsequently have higher ED utilisation [58, 59].

When participants are observed over a substantial period of time (for example over 6 to12 months), there is a risk that association between the predisposing risk factor(s) and outcome (unplanned ED presentation) may change [60, 61]. In people diagnosed with cancer, stage of disease is likely to change; they will either no longer have an active cancer diagnosis or will progress to an advanced or metastatic stage of the disease. No studies included in this review included stage of disease as a time varying variable, a potentially important consideration that is related to the outcome of interest. This was demonstrated in a study by Minami et al. where stage of disease was recorded at diagnosis only. The authors reported that patients who presented to the ED were more likely to be hospitalised, have a longer length of hospital stay, and were more likely to have bony metastases. Patients were followed for an average 242 days (until death). Because stage of disease was only recorded at diagnosis, and it is likely that patients with early stage of disease progressed, the incidence of unplanned ED presentations associated with metastatic disease may have been underestimated [40].

### External validity

#### Setting

The healthcare setting in which studies are conducted may have substantial influence on study outcomes and external validity [62]. For example, Harrison et al. [39] reported that only 15 (14%) patients in their study, who received SACT made an unplanned ED visit, however 31 (29%) had an unplanned oncologist visit and eight (7.5%) an unplanned hospitalisation. Two studies [36, 47] provided clear description of usual care, and described access to phone support for patients who develop SACT related toxicities, or who need access to unplanned healthcare. This service may have significantly impacted need for or decision to attend ED. While it is not possible to statistically control for variance in healthcare settings and resultant ED presentations, they should at least be described in enough detail to allow readers to fully appreciate contextual factors of study participants that may or may not influence ED utilisation [62].

Another important consideration when judging external validity is the country and broader healthcare system in which studies are undertaken. While healthcare settings may appear similar, there are important population differences that affect generalisability. For example, all of the studies included in this review were undertaken in developed countries, but demographic differences including racial disparities, population age, and access to cancer screening could affect the external validity of study results [62–64]. There is variation in access to adequate health care within and between countries because of fundamental differences in the way healthcare systems are designed and delivered, as well as sociocultural factors that impact access to health care [63]. For example, a US study by Whitney et al. [65] identified that unplanned ED presentations in the first year after cancer diagnosis occurred most commonly in persons of non-Hispanic black race/ethnicity. Additionally, as socioeconomic status decreased, the rate of unplanned ED presentations increased.

The majority of studies only reported data from participants with one specific cancer type, limiting generalisability of these findings to that specific cohort of cancer patients. Similarly, few studies described the range of SACTs administered to participants, and other medications that might be prescribed to minimise occurrences of treatment related toxicities, for example Colony Stimulating Factors and anti-emetic regimens.

## Implications for research/practice

Observational studies offer an efficient method to measure the incidence of and risk factors associated with unplanned ED presentations among patients receiving SACT. However, there is substantial diversity in study populations and in the methodological approaches used to measure incidence meaning findings cannot be synthesised. There are several methodological limitations in published studies that need to be addressed to strengthen potential of future initiatives to reliability inform practice. The STROBE checklist offers an important source of quality for the reporting of observational studies. In addition, we offer recommendations on how to apply the STROBE checklist in this unique group of people. These include:

1. To accurately identify the incidence of ED presentations, participants should be enrolled at diagnosis or commencement of SACT, and details of prior exposure to anti-cancer treatments should be described. Where possible, efforts should be made to follow participants until treatment discontinuation, or death [66, 67].
2. A detailed description of the study setting should be reported. Specifically, the health service and the services available to support people receiving SACT who may develop toxicities away from the healthcare setting is essential for generalisability of study findings [68].
3. Data for time varying covariates such as age, comorbidities, and stage of disease should be collected at each event (unplanned ED presentation) that occurs and accounted for as time-varying covariates during statistical analyses [69].
4. As reasons for loss to follow up are likely to be related to the exposure or the outcome, detailed descriptions of demographic differences of participants lost to follow up should be reported [54].
5. The outcome should be clearly defined, and crude numbers of individuals and the number of visits made should be reported. Where possible, modelling for recurring events should be included in statistical analysis [49].

## Limitations

This review is limited by the exclusion of unpublished and grey literature. This omission could have reduced the number of studies included in this review and subsequently impacted the findings and conclusions. Unpublished literature was omitted because of the intention of the review, that is, to review the methods used, and assess the methodological rigor of studies. Literature published in languages other than English were excluded from this review and may also have impacted the number of studies included in this review. We also acknowledge that there are other tools to conduct a risk of bias assessment that may have produced different results than the results reported in this review.

## Conclusion

There is substantial methodological diversity in literature that reports the incidence of unplanned ED presentations by cancer patients receiving SACT, making it difficult to accurately estimate and understand the incidence of unplanned ED presentations in this population. The internal validity of the studies included in this review was limited by study design, inadequate description of participants who were lost to follow up, differences in outcome definition and measurement, and variance in factors that were identified as potential confounders. External validity was hindered by differences in the description of the setting and regular care provided to cancer patients receiving SACT who develop symptoms, as well as important differences in demographics such as age and diagnosis.

Unplanned ED presentations by patients receiving SACT could be viewed as an indicator of quality cancer care. The development of local policy and targeted interventions to mitigate unplanned ED presentations should reflect local need as well as available resources. Therefore, it is imperative that future research is designed and reported by means that allows clinicians and policy makers to judge the generalisability of the findings.

## Supplementary Information

**Additional file 1: Table 1.** Search strategy in three databases.

**Additional file 2.** Risk of Bias tools.

**Additional file 3.** Data extraction spreadsheet.

**Additional file 4: Table 1.** Additional analysis of included studies.

## Data Availability

All data generated or analysed during this study are included in this published article [and its supplementary information files].

## Abbreviations

SACT: Systemic anti-cancer therapy
ED: Emergency department
SWiM: Synthesis without meta-analysis
NOS: The Newcastle Ottawa Scale
ESAS: Edmonton Symptom Assessment Scale
