# Supplementary material for: Methodological approaches to measuring the incidence of unplanned emergency department presentations by cancer patients receiving systemic anti-cancer therapy: a systematic review
Source: BMC Med Res Methodol. 2022 Mar 21;22:75. doi: 10.1186/s12874-022-01555-3 (PMC8935762; doi:10.1186/s12874-022-01555-3)
Supplement: Supplementary file 1 — Additional file 1: Table 1. Search strategy in three databases. [file 12874_2022_1555_MOESM1_ESM.docx]

Table 1 Search strategy in three databases

| CINAHL 01 11 19 – multipurpose search | | |
| --- | --- | --- |
| 1 | (MH "Emergency Service+") | 53778 |
| 2 | emergency department* OR emergency service* OR "accident and emergency".mp | 101638 |
| 3 | (ED OR ER OR “emergency room” OR “emergency care” OR emergency) N3 present*.mp | 97113 |
| 4 | (MH "Antineoplastic Agents+") | 102679 |
| 5 | chemotherap* OR anticancer OR anti-cancer OR antineoplastic.mp | 121537 |
| 6 | (cancer* OR oncology OR neoplasm*) N4 (patient* or client* or people or women or men or male* or female*) | 399596 |
| 7 | 1 OR 2 OR 3 AND 4 OR 5 AND 6 | 298 |
| 8 | 2010-2019 | 258 |
| 9 | Downloaded for further review after initial title screen | 47 |

| MEDLINE 29 10 19 – multipurpose search | | |
| --- | --- | --- |
| 1 | exp Emergency Service, Hospital/ | 73652 |
| 2 | ((emergency department* or emergency service* or "accident and emergency" or ED or ER or emergency room or emergency care or emergency) adj3 present*).mp | 22969 |
| 3 | exp antineoplastic agents/ | 1072457 |
| 4 | chemotherap* or anticancer or anti-cancer or antineoplastic.mp | 772047 |
| 5 | (cancer* OR oncology OR neoplasm*) adj4 (patient* or client* or people or women or men or male* or female*).mp | 448026 |
| 6 | 1 OR 2 AND 3 OR 4 AND 5 | 151 |
| 7 | 2010-2019 - results screened based on title and abstract | 122 |
| 8 | Downloaded for further review after initial title screen | 31 |

| EMBASE 04 11 19 – multipurpose search | | |
| --- | --- | --- |
| 1 | exp emergency ward/ | 135891 |
| 2 | emergency department* or emergency service* or (accident adj2 emergency") or ED or emergency room or emergency care or emergency.mp. | 579885 |
| 3 | exp antineoplastic agent/ | 2244297 |
| 4 | chemotherap* or anticancer or anti-cancer or antineoplastic.mp | 1192914 |
| 5 | (cancer* OR oncology OR neoplasm*) adj4 (patient* or client* or people or women or men or male* or female*).mp | 853099 |
| 6 | 1 OR 2 AND 3 OR 4 AND 5 | 3396 |
| 7 | 2010-2019 | 2912 |
| 8 | Downloaded for further review after initial title screen | 290 |
