# Supplementary material for: Methodological approaches to measuring the incidence of unplanned emergency department presentations by cancer patients receiving systemic anti-cancer therapy: a systematic review
Source: BMC Med Res Methodol. 2022 Mar 21;22:75. doi: 10.1186/s12874-022-01555-3 (PMC8935762; doi:10.1186/s12874-022-01555-3)
Supplement: Supplementary file 2 — Additional file 2. Risk of Bias tools. [file 12874_2022_1555_MOESM2_ESM.docx]

Risk of Bias tools

**NEWCASTLE - OTTAWA QUALITY ASSESSMENT SCALE**

**COHORT STUDIES**

Note: A study can be awarded a maximum of one star for each numbered item within the Selection and Outcome categories. A maximum of two stars can be given for Comparability

**Selection**

1) Representativeness of the exposed cohort

a) truly representative of the average _______________ (describe) in the community ****

b) somewhat representative of the average ______________ in the community ****

c) selected group of users eg nurses, volunteers

d) no description of the derivation of the cohort

2) Selection of the non exposed cohort

a) drawn from the same community as the exposed cohort ****

b) drawn from a different source

c) no description of the derivation of the non exposed cohort

3) Ascertainment of exposure

a) secure record (eg surgical records) ****

b) structured interview ****

c) written self report

d) no description

**Comparability**

1) Comparability of cohorts on the basis of the design or analysis

a) study controls for _____________ (select the most important factor) ****

b) study controls for any additional factor **** (This criteria could be modified to indicate specific control for a second important factor.)

**Outcome**

1) Assessment of outcome

a) independent blind assessment ****

b) record linkage ****

c) self report

d) no description

2) Was follow-up long enough for outcomes to occur

a) yes (select an adequate follow up period for outcome of interest) ****

b) no

3) Adequacy of follow up of cohorts

a) complete follow up - all subjects accounted for ****

b) subjects lost to follow up unlikely to introduce bias - small number lost - > ____ % (select an adequate %) follow up, or description provided of those lost) ****

c) follow up rate < ____% (select an adequate %) and no description of those lost

d) no statement

**NEWCASTLE - OTTAWA QUALITY ASSESSMENT SCALE**

**CROSS-SECTIONAL**

**Selection:** (Maximum 5 stars)

1. Representativeness of the sample:
2. Truly representative of the average in the target population. * (all subjects or random sampling)
3. Somewhat representative of the average in the target population. * (non-random sampling)
4. Selected group of users.
5. No description of the sampling strategy.

2) Sample size:

1. Justified and satisfactory. *
2. Not justified.

3) Non-respondents:

1. Comparability between respondents and non-respondents characteristics is established, and the response rate is satisfactory. *
2. The response rate is unsatisfactory, or the comparability between respondents and non-respondents is unsatisfactory.
3. No description of the response rate or the characteristics of the responders and the non-responders.

4) Ascertainment of the exposure (risk factor):

1. Validated measurement tool. **
2. Non-validated measurement tool, but the tool is available or described.*
3. No description of the measurement tool.

**Comparability:** (Maximum 2 stars)

1) The subjects in different outcome groups are comparable, based on the study design or analysis. Confounding factors are controlled.

1. The study controls for the most important factor (select one). *
2. The study control for any additional factor. *

**Outcome:** (Maximum 3 stars)

1) Assessment of the outcome:

1. Independent blind assessment. **
2. Record linkage. **
3. Self report. *
4. No description.

2) Statistical test:

1. The statistical test used to analyze the data is clearly described and appropriate, and the measurement of the association is presented, including confidence intervals and the probability level (p value). *
2. The statistical test is not appropriate, not described or incomplete.
