# Supplementary material for: Methodological approaches to measuring the incidence of unplanned emergency department presentations by cancer patients receiving systemic anti-cancer therapy: a systematic review
Source: BMC Med Res Methodol. 2022 Mar 21;22:75. doi: 10.1186/s12874-022-01555-3 (PMC8935762; doi:10.1186/s12874-022-01555-3)
Supplement: Supplementary file 3 — Additional file 3. Data extraction spreadsheet. [file 12874_2022_1555_MOESM3_ESM.docx]

Additional file 3

Data extraction spreadsheet

|  |  | **Methods** | | | | | | | | | | |
| --- | --- | --- | --- | --- | --- | --- | --- | --- | --- | --- | --- | --- |
|  | Objective | Study design | | Setting | | | | | Participants | | | |
| **Author** | Study objective | Study design | Single / multi-site /population | | Country | Dates of recruitment | Period of follow-up | Regular care description | Inclusion criteria | Tumour stream | Stage of disease | Type of treatment |
|  |  |  |  | |  |  |  |  |  |  |  |  |

| **Methods** | | | | | | | | | | |  |
| --- | --- | --- | --- | --- | --- | --- | --- | --- | --- | --- | --- |
|  | Variables | | | | | | | | Statistical methods | |  |
| **Author** | Exposure | Enrolment triggered by | Period of observation | Cause of ED presentation | Method to identify treatment related ED presentations | Description of outcome | Confounders adjusted for | Stage as confounder | Statistical methods | Time varying covariates | |
|  |  |  |  |  |  |  |  |  |  |  | |

| **Results** | | | | | |
| --- | --- | --- | --- | --- | --- |
|  | Study results | | | | |
| **Author** | Sample size | Age mean (SD) | ED presentations | ED visits per person | Loss to follow up |
|  |  |  |  |  |  |
