# Supplementary material for: Methodological approaches to measuring the incidence of unplanned emergency department presentations by cancer patients receiving systemic anti-cancer therapy: a systematic review
Source: BMC Med Res Methodol. 2022 Mar 21;22:75. doi: 10.1186/s12874-022-01555-3 (PMC8935762; doi:10.1186/s12874-022-01555-3)
Supplement: Supplementary file 4 — Additional file 4: Table 1. Additional analysis of included studies. [file 12874_2022_1555_MOESM4_ESM.docx]

Additional file 4

Table 1 Additional analysis of included studies

| **Authors** | **Population** | | **Enrolled at diagnosis or initiation of SACT** | **Observation period** | | | **Follow-up period** | | **Summary of follow-up time** | **Reasons for loss to follow-up** | | | **ED presentations per participant case** |
| --- | --- | --- | --- | --- | --- | --- | --- | --- | --- | --- | --- | --- | --- |
| Baenda-Canada et al. | Diagnosed with breast cancer, receiving (neo)adjuvant or palliative chemotherapy | | x |  | | |  | |  |  | | | 0.14 ED visits per participant case |
| Barbera et al. | Aged >= 18 years, diagnosed with stage I-III breast cancer, received adjuvant therapy within 6 months of diagnosis | | x | first day of chemotherapy to 30 after last day of chemotherapy | | |  | |  |  | | |  |
| Colligan et al. | Diagnosed with breast, lung, lymphoma or colorectal cancer, medicare fee-for-service patients, with both Part A and Part B coverage, served by the COME HOME and PCCP programs | |  | 28 days | | | 3 years, death or end of study period | | Aprox one third of participants died during study period | Tumour stream only | | |  |
| Dufton et al. | Aged >= 18 years, diagnosed with cancer, receiving SACT in the outpatient setting during study dates | |  | 28 days | | |  | |  |  | | | 0.88 ED visits per participant case |
| Enright et al. | Aged >18, diagnosed with stage I to III EBC and received at least one cycle of adjuvant chemotherapy within 4 months of breast cancer surgery date | x | | | 30 days |  | |  | | |  | 0.35 ED visits per participant case | |
| Eskander et al. | Aged >18 years, diagnosed with malignancies of the oral cavity, oropharynx, larynx and hypopharynx | x | | | 90 days | For up to 90 days from date when last treatment modality was completed | | Total days at risk 146,422 | | |  | 1.13 ED visits per participant case | |
| Fisher et al. | Aged 18-64 years, diagnosed with early or metastatic breast, metastatic lung, metastatic CRC, or NHL, CLL | x | | | During 12 month follow up and up to 30 days after | 12 months after index date | | 2.2 years | | | 77% of patients had 12 months of follow-up data 1.4% of patients died within a year of the index date | Physician office 0.30, Hospital outpatient 0.8 ED visits per participant case | |
| Harrison et al. | Aged >=21, diagnosed with NHL, breast, CRC, NSCLC, H&N cancer | x | | | Post cycle 1 | 1 cycle | |  | | | NA | NA* | |
| Hoverman et al. | Aged >= 18 years. Diagnosed with breast, CRC or lung cancer, treated by TXO physician | x | | | 30 days |  | | Average time on treatment 105-106 days | | |  | 0.21 ED visits per participant case (baseline)  0.11 ED visits per participant case (Innovent) | |
| Kamat et al. | 1L or 2L chemotherapy for advanced bladder cancer | x | | | Time from first dose of chemotherapy through the end of the last cycle in that line of therapy | Minimum 24 months, followed until earliest date of death, health maintenance enrolment, last known date of follow-up in database, or end of the study period | | 1st line treatment - 15.8 weeks 2nd line treatment 12.8 weeks | | |  | 0.8 ED visits per participant case(1st line treatment)  0.6 ED visits per participant case (2nd line treatment) | |
| Korykowsky et al. | Aged >=18 years, advanced NSCLC | x | | | Until March 2017 |  | | 16.9 months pre-IO and 8.9 months post I) | | |  | 0.77 ED visits per participant case (pre-IO)  0.66 ED visits per participant case (post-IO) | |
| Li et al. | Post menopausal HR+/HER2- mBC who discontinued NSAI and began evorlimus or chemotherapy | x | | |  |  | |  | | |  | 0.10 ED visits per person (line 1) | |
| Livingstone et al. | Newly diagnosed with cancer receiving chemotherapy in out-patient clinics | x | | | during study period (12 months) |  | |  | | |  | 0.57 ED visits per participant case | |
| Mehra et al. | Docetaxel for advanced prostate cancer | x | | | Monthly | Until loss to follow-up or censor date | | 15.82 months | | |  |  | |
| Minami et al. | Diagnosed with lung cancer, died of lung cancer during the 3 year study period, and followed up until diagnosis | x | | | during chemotherapy | Until death | |  | | | Death |  | |
| Peyrony et al. | Diagnosed with cancer, received ICB during study period |  | | | throughout study (5 years) | Only first visit (ED, or hospitalisation) in dataset counted | | NA | | | NA | NA* | |
| Pittman et al. | Received at least one cycle of curative chemotherapy for stages I-III  breast cancer |  | | | 30 days |  | |  | | |  | 0.89 ED visits per participant case | |
| Schwartzberg et al. | Aged 18-70 years. Diagnosed with a solid tumour and receiving therapy with anthracycline plus cyclophosphamide, cisplatin, or carboplatin | x | | | during chemotherapy | At least 1 month post index date | | 8.9 months | | |  | 0.19 ED visits per participant case | |
| Tang et al. | Diagnosed with stage I-III breast cancer, receiving curative, adjuvant or neoadjuvant chemotherapy |  | | | 30 days |  | |  | | |  | 0.62 ED visits per participant case | |
| Ward et al. | Aged >=18 years, undergoing chemotherapy for breast, CRC or NSCLC |  | | | 6 months | Until patients withdrew, ceased chemotherapy and did not recommence within 30 days, died, or the census date was reached | | Presented in figure | | |  | 0.74 ED visits per participant case | |
| Williams et al. | Aged >=65 years, female, stage I-III breast cancer, receiving chemotherapy, hormone, or targeted therapy | x | | | start of cancer treatment until death or censorship | Until death or censorship | |  | | |  |  | |
| *ED presentations per participant case unable to be calculated as study only reports the number of unique individuals that made on or more ED presentation and not the total number of ED presentations made.  ED – emergency department, COME HOME - Community Oncology Medical Home, PCCP - Patient Care Connect Program, SACT – systemic anti-cancer therapy, EBC – early breast cancer, CRC – colorectal cancer, NHL – non-Hodgkin’s lymphoma, CLL – chronic lymphocytic leukaemia, NSCLC – non-small lung cancer, H&N – head and neck, TXO – Texas Oncology, HR+/HER2- - hormone receptor positive/ human epidermal growth factor negative, mBC – metastatic breast cancer, ICB – immune checkpoint blockade, 1L – first line, 2L – second line | | | | | | | | | | | | | |
